# Effects of Virtual Reality-Based Relaxation Techniques on Psychological, Physiological, and Biochemical Stress Indicators

**DOI:** 10.3390/healthcare9121729

**Published:** 2021-12-14

**Authors:** Eglė Mazgelytė, Virginija Rekienė, Edita Dereškevičiūtė, Tomas Petrėnas, Jurgita Songailienė, Algirdas Utkus, Gintaras Chomentauskas, Dovilė Karčiauskaitė

**Affiliations:** 1 Department of Physiology, Biochemistry, Microbiology and Laboratory Medicine, Institute of Biomedical Sciences, Faculty of Medicine, Vilnius University, LT-03101 Vilnius, Lithuania; dovile.karciauskaite@mf.vu.lt; 2 Human Study Center, LT-01132 Vilnius, Lithuania; virginija.rekiene@gmail.com (V.R.); training@humanstudy.lt (E.D.); gintaras@humanstudy.lt (G.C.); 3 Department of Human and Medical Genetics, Faculty of Medicine, Institute of Biomedical Sciences, Vilnius University, LT-03101 Vilnius, Lithuania; tomas.petrenas@mf.vu.lt (T.P.); jurgita.songailiene@mf.vu.lt (J.S.); algirdas.utkus@mf.vu.lt (A.U.)

**Keywords:** stress, virtual reality, relaxation, biofeedback

## Abstract

Various relaxation techniques could benefit from merging with virtual reality (VR) technologies, as these technologies are easily applicable, involving, and user-friendly. To date, it is unclear which relaxation technique using biofeedback combined with VR technology is the most effective. The study aimed to compare the effectiveness of brief VR-based biofeedback-assisted relaxation techniques including electroencephalographic biofeedback, mindfulness-based biofeedback, galvanic skin response biofeedback, and respiratory biofeedback. Forty-three healthy volunteers (age 34.7 ± 7.2 years), comprising 28 (65%) women and 15 (35%) men, were enrolled in the study. All the participants were exposed to four distinct relaxation sessions according to a computer-generated random sequence. The efficacy of relaxation methods was evaluated by examining psychological, physiological, and biochemical stress indicators. All VR-based relaxation techniques reduced salivary steroid hormone (i.e., cortisol, cortisone, and total glucocorticoid) levels and increased galvanic skin response values. Similarly, all interventions led to a significantly reduced subjectively perceived psychological strain level. Three out of the four interventions (i.e., electroencephalographic, respiratory, and galvanic skin response-based biofeedback relaxation sessions) resulted in a decreased self-reported fatigue level. We suggest that newly developed VR-based relaxations techniques are potential tools for stress reduction and might be particularly suitable for individuals who are not capable of adhering to a strict and time-consuming stress management intervention schedule.

## 1. Introduction

Various relaxation techniques that include practices such as progressive muscle relaxation, deep breathing exercises, and mindfulness meditation are used to relax and reduce a person’s stress and anxiety. The short-term physiological relaxation response is regulated by two branches of the autonomic nervous system (ANS). The parasympathetic branch of the ANS is more active while resting and therefore, its activation reduces physiological arousal. The sympathetic branch of ANS is more active during emergency reactions and promotes physiological arousal [1]. Relaxation is the ability of the human body to reduce the physiological arousal that manifests itself in the human body through a less activated sympathetic branch, decreased muscle tension, diminished heart rate, lowered respiration rate, increased skin temperature, and decreased sweating [2].

Various relaxation techniques use different instructions and training to elicit a relaxation response. For example, a deep breathing exercise procedure is based on breathing at controlled rates, particularly at 6 breaths/minute [3], whereas when applying a cognitive relaxation technique, people are asked to engage in relaxation strategies such as reliving one’s pleasant memories or replaying one’s favorite song internally for a period of 15–20 min [4]. Mindfulness meditation strategies refer to focusing one’s attention in a non-judgmental manner to experiences that exist in the present moment—this may include focusing on breath, body scan, and mindful walking [5,6]. All these practices have a similar goal—to produce one’s body’s relaxation responses that are characterized by diminished heart rate or slower breathing and the psychological effects of calmness. Relaxation biofeedback training, which is based on altering one’s physiological activity (e.g., muscle tension, heart rate, blood flow) using visual, auditory (or both) feedback is found to be a very effective tool in reducing a person’s stress and anxiety level on a daily basis [7,8]. Moreover, biofeedback training techniques have several advantages, as these methods are non-invasive training tools that enable individuals to observe their physiological body signals and to gain voluntarily control of autonomous physiological body signals and objectively observe the progress of relaxation. When it comes to the length of time of relaxation, some researchers [9] suggest that relaxation longer than 29 min is associated with improved psychological well-being. Other studies [10,11] indicate that brief relaxation sessions (12-min or 13-min) that are practiced especially by the inexperienced meditators are associated with decreased anxiety and diminished negative mood [10], and also with better cognitive functioning and emotional well-being [11]. To summarize, short relaxation sessions that are enjoyable, practical, and easily replicable are beneficial for psychological well-being, especially for the inexperienced meditators.

A newly developed virtual reality (VR) technology is considered as a promising tool in reducing stress level and coping with anxiety [12,13,14]. Dellazizzo et al. [15] conducted a meta-review of 11 meta-analyses examining the efficacy of VR-based interventions for various psychiatric disorders and concluded that, comparing with inactive controls, VR-based techniques significantly improved neurocognitive and anxiety-related disorders, but there were no significant differences between VR-based and standard evidence-based approaches. Similar results were described in the meta-analysis investigating the efficiency of virtual reality exposure therapy (VRET) for post-traumatic stress disorder (PTSD): comparing with waitlist controls, VRET significantly alleviated PTSD symptom severity and improved depressive symptoms; however, the comparison of VRET and active control group (traditional exposure therapy) yielded non-significant results [16]. VR interventions were found to be an effective distraction tool for the management of pain and anxiety associated with medical procedures [17,18]. Recently published meta-analysis showed that VR technique was superior to the standard care methods in decreasing pain scores in the pediatric population exposed to needle-related medical procedures [17]. Moreover, a meta-analysis conducted by Eijlers et al. [18] revealed that VR was more effective in reducing pain and anxiety during different types of medical procedures (venous access, dental, burn or oncological care, exposure before elective surgery under general anesthesia) compared with standard care method in a group of children and adolescents.

Diverse relaxation techniques could benefit from merging with virtual reality technologies, as these technologies have several additional advantages: they are easily applicable, involving, user-friendly, and they provide wide possibilities for testing, training, and treatment. In addition, VR technologies could be characterized as tools of accurate control and measurement [19]. VR technologies enable the full involvement of the person in the intended experiences, as they separate them from unwanted disturbing external stimuli by effectively controlling visual and audio input by use of a VR headset (which is not possible while using a computer and a monitor) [20,21]. However, it is unclear which relaxation technique using biofeedback combined with VR technology is the most effective.

Although meta-analyses revealed the effectiveness of various relaxation techniques including transcendental meditation [22], mindfulness-based meditations [23], and multimodal biofeedback methods [24], it is unknown what specifically in these methods affects a person’s stress and anxiety reduction. Relaxation methods in a variety of settings are applied differently, and they are often adjusted to the progress of an individual, and the results are dependent on the competence of the instructor and the participant. In the context of a great variety of relaxation methods, it remains unclear whether different relaxation methods are based on the distinct or on the same biological relaxation mechanism. Thus, it is difficult to determine which elements of relaxation methods are the most effective: modality of the relaxation technique, duration of the relaxation session, the instructor’s personality and competence. Moreover, the effectiveness of the applied relaxation method is usually assessed using only self-assessment questionnaires [22]. The use of questionnaires in stress research is a well-established tradition, mainly for simplicity of application. However, self-assessment psychological questionnaires are subjective and inaccurate, and they are based on a person’s ability to reflect changes in one’s physical, emotional, thinking, and behavioral stress symptoms and, therefore, they cannot objectively measure changes in stress level.

Biofeedback-based relaxation methods have been extensively studied and proven to be effective in activating the parasympathetic nervous system [24]. Assessment of the biomarkers of the autonomic nervous system allows a more objective evaluation of the efficiency of distinct relaxation methods. Physiological stress indicators, such as breathing rate, heart rate and its variability, galvanic skin response, and changes in brain wave activity are usually taken into account.

The biochemical response to stress is regulated through the activation of the hypothalamic-pituitary-adrenal (HPA) axis. Increased HPA axis activity can be observed by measuring the instantaneous concentration of cortisol in a person’s saliva [25]. Cortisol is a biochemical stress marker, and changes in cortisol levels between pre- and post-relaxation intervention can be measured objectively. Monitoring a person’s stress level using biochemical stress indicators is not only a promising innovative method, but it could also be considered as the main technique for measuring the efficacy of VR-based relaxation sessions.

Research conducted so far does not provide enough data to develop a reliable relaxation method which works without the help—or with minimal help—of a qualified instructor. Therefore, this study aimed to examine the impact of distinct VR-based relaxation techniques on psychological, physiological, and biochemical stress indicators. We hypothesized that different biofeedback-assisted virtual reality-based relaxation sessions will have distinct effects on the average heart rate, galvanic skin response (digitized resistance) values, instantaneous concentration of cortisol in the saliva, as well as psychological strain.

## 2. Materials and Methods

### 2.1. Study Participants

Forty-three apparently healthy volunteers (age 34.7 ± 7.2 years), comprising 28 (65%) women and 15 (35%) men, were enrolled in the study. Participants were recruited at the Human Study Center via a pre-registration form. Each enrolled individual was contacted by the researchers by telephone. Potential subjects were excluded for medical conditions including chronic heart disease, metabolic and endocrine disorders, as well as mental diseases. Moreover, individuals were excluded if they were taking steroid hormone medications such as hydrocortisone, prednisone, and dexamethasone. Participants provided written informed consent before entering the study. The study protocol was approved by the Lithuanian Bioethics Committee (No. 2019/5-1135-626). The procedures used in this study adhere to the tenets of the Declaration of Helsinki. Table 1 reports the sociodemographic and lifestyle characteristics of the study sample.

### 2.2. Experimental Design

To evaluate the change of psychological and physiological stress indicators, as well as biochemical stress markers, from pre- to post-single application of relaxation techniques, the team of experts of the Human Study Center and Vilnius University carried out the study. The study used a within-subject crossover design. Individuals were required to attend the Human Study Center on five separate occasions, with a minimum of 1 day between sessions and a maximum of 21 days between sessions. Sessions occurred between 9:00 a.m. and 05:45 p.m. with the majority of sessions (74.4%) being held in the afternoon. The flexible session time was chosen as convenient and allowing participants to make their choice about session time thus bringing the session time conditions in research close to practical application of procedures in real life. Participants were exposed to four distinct 12-min virtual reality-based biofeedback-assisted relaxation techniques, including electroencephalographic (EEG) biofeedback, mindfulness-based biofeedback, galvanic skin response (GSR) biofeedback, and respiratory biofeedback. The order of administration of these conditions was randomized using a computer-generated random sequence. The choice of 12-min session duration was based on our findings in pilot research in which many not trained individuals indicated that during longer duration sessions of 20 min, it was difficult for them to maintain attention on VR content and feedback.

### 2.3. Experimental Procedure

During the first visit in the Human Study Center, individuals received explanations about the experimental procedure and signed written informed consent. Additionally, subjects completed a questionnaire on sociodemographic and lifestyle characteristics, filled out Perceived Stress Scale (PSS) and State-Trait Anxiety Inventory (STAI). Then, participants were asked to rate their mood status, fatigue, and strain level. Additionally, the first saliva sample was collected. Following that, the instructor attached the sensors on the subjects’ left ear and on the two fingers of the left hand to monitor heart rate and conductivity of the skin. Participants were also asked to sit comfortably on a chair, and to keep their left arm still on their left thigh during the entire biofeedback training session period. Before starting the EEG, GSR, and mindfulness-based biofeedback-assisted relaxation session, individuals were asked to lower their arousal by any means helpful for them, e.g., by means of acting on their disturbing thoughts, emotions, body sensations, or any visual (imagery). In the instruction before the respiratory biofeedback-assisted session, individuals were instructed on how to focus on their breathing, and they were asked to breath slowly and evenly. After the verbally provided instructions, the 12-min relaxation session started. Physiological stress measures were recorded during the first and the last minute of relaxation session. After the session, subjects were again asked to evaluate their mood status, fatigue, and strain level. The second saliva sample was collected immediately after the relaxation session. The average duration of participants’ presence in the Human Study Center laboratory during five distinct occasions ranged from 30 to 45 min.

### 2.4. Description of Distinct Relaxation Sessions

#### 2.4.1. EEG Biofeedback

During the EEG biofeedback-assisted relaxation session, virtual reality headsets Oculus Rift, manufactured by Oculus VR in USA, were used for every individual to provide visual as well as acoustic feedback connected to a Muse EEG device, manufactured by InteraXon Inc. in Canada, that provided information about the level of the person’s alpha brainwave activity. The Muse EEG headband has electrodes located analogous to Fpz, AF7, AF8, TP9, and TP10 with electrode Fpz used as the reference electrode [26]. These devices work well in pair for the EEG feedback and data collection. The Muse EEG band has 7 open electrodes that contact with the user’s skin—5 on the forehead and 2 behind the ears. Additionally, the headband is slick enough to fit comfortably together with this particular VR headset and not being influenced by it. The selected indicator of relaxation and feedback in the current study was alpha wave 8–12 Hz dominance (%) over other brainwaves. The alpha wave is widely used as an indicator of deep relaxation during meditation [27].

Individuals were informed via the text seen in the virtual reality headsets that they will see a tropical island with heavy rains, thunder and a storm, and depending on how they manage to lower their arousal level (to relax), and they will see changes in weather on the island depending on alpha percentage change. Both visual and acoustic feedback informed the person that his/her arousal was going down. The lesser the arousal level of the person, the more positive changes the person observed on the island—less rain, wind, storm and thunder, and more sun, birds chirping. Additionally, numerical indication of percentage of alpha waves was provided on VR headset.

#### 2.4.2. Mindfulness-Based Biofeedback

During the mindfulness-based biofeedback relaxation session, Oculus Rift virtual reality headsets were used for every individual to provide visual feedback on their ability to focus their attention. Individuals were informed via text in the virtual reality headset that they will see a blue dot that changes its position depending on a person’s head movements. The task was to transform moving spheres into objects, while the transformation happened if one could concentrate his/her attention (via a blue dot) for 10 s on slowly moving spheres scattered in the static background of the photographed view of a city park. In case of successful concentration on the chosen spheres, the sphere itself began to transform into an object—a butterfly, a shell, etc. After its final transformation, the object disappeared. The goal was to animate as many objects as possible.

#### 2.4.3. GSR Biofeedback

During the galvanic skin response biofeedback-assisted relaxation session, Oculus Rift virtual reality headsets were used to provide visual as well as acoustic feedback connected to the GSR device that provided information about the level of person’s electrodermal activity (digitized resistance). In the instruction seen in the virtual reality headsets, individuals were asked to lower their arousal by any means helpful to them, e.g., by acting on their thoughts, emotions, body sensations, or any visual (imagery) means. Individuals were informed that via the virtual reality headsets they will see a tropical island with heavy rains, thunder, and storm, and depending on how they manage to lower their arousal level (to relax), they will see changes in weather on the island. Both visual and acoustic feedback informed the person that his/her arousal is going down. The less the arousal level of the person was, the more positive were the changes the person observed in the environment on the island—less rain, wind, storm and thunder, and more sun, birds chirping.

#### 2.4.4. Respiratory Biofeedback

During the respiratory biofeedback-assisted relaxation session, Oculus Rift virtual reality headsets were used to provide visual feedback of the subject’s ability to breath in a slow, even, and diaphragmatic manner. In the present study, a stretch belt was used in the abdominal area and it measured the tension during inhalation and exhalation during the breathing cycle. The repeating breathing rhythm over time was presented to a person in a sine wave. All individuals saw two lines via virtual reality headsets: the red one—heart rate’s wave and blue one—respiratory wave, in a background of a night sky with stars. Individuals were instructed via the virtual reality headsets that they will see the thin blue line—their respiratory wave that will be changing, depending on how they breath (while inhaling, the wave will rise, while exhaling, the wave will fall). Furthermore, individuals were instructed that they will see the red line that will represent their heart rate. Finally, individuals were instructed that depending on the lowering level of their arousal, their respiratory wave will be getting slope and smooth, as well as their heart rate line might start to fluctuate synchronously with their respiratory line, showing sinus arrythmia.

### 2.5. Measures

#### 2.5.1. Perceived Stress and Anxiety

Perceived Stress Scale (PSS) was used as a measure of the degree to which an individual has perceived life as unpredictable, uncontrollable, and burdensome. The 10-item version indicated participants’ stress perception over the past month. Participants were asked to rate each item on a Likert-type response scale ranging from 1 = never to 5 = very often. Higher overall score indicates a greater perceived stress level.

State and Trait Anxiety subscales of the STAI were used as a subjective measure of anxiety. The S-Anxiety scale evaluates how respondents feel “right now” and the T-Anxiety scale assesses how subjects “usually” feel. Each STAI score ranges from 20 to 80, with higher scores indicating greater state and trait anxiety levels [28].

#### 2.5.2. Mood Status, Fatigue, and Strain

Before and after each relaxation session, participants were asked to rate their mood status, fatigue, and strain using a five-point 3 Likert scales developed by the Human Study Center; 1 point indicated depressed mood, high fatigue, and strain, while 5 points indicated good mood, low fatigue, and low strain level.

#### 2.5.3. Salivary Cortisol and Cortisone Levels

Each participant provided two saliva samples using Salivette^®^ (Sarstedt, Rommelsdorft, Nümbrecht, Germany) devices before and immediately after each experimental session (pre- and post-session samples). The subjects were asked to restrain from alcohol consumption for 48 h, intense exercise for 24 h, eating, drinking (except water), smoking, and brushing their teeth or using dental floss for 1 h prior to each saliva collection procedure. Saliva samples were stored at −80 °C until the analysis. The samples were treated in the sequence of centrifugation for 10 min at 4000 rpm, liquid–liquid extraction with ethyl acetate, and resuspension in methanol/water containing 0.1% formic acid in a ratio of 50:50 (*v*/*v*). The chromatographic separation was performed on the ultra-high performance liquid chromatography (UHPLC) system, which consisted of two Shimadzu LC-30AD binary pumps, a Shimadzu SIL-30AC autosampler and a Shimadzu CTO-20AC column oven (Shimadzu Corporation, Kyoto, Japan). The UHPLC was coupled to Shimadzu LCMS-8060 triple quadrupole tandem mass spectrometer equipped with an electrospray ionization source (Shimadzu Corporation, Kyoto, Japan) which was operated in the positive ionization mode. For each analyte, two ion pairs were selected with the most sensitive transition being used for quantification, and the rest for confirmation. The UHPLC column was Poroshell 120, EC-C18, (3.0 × 75 mm, 2.7 μm) column (Agilent Technologies, Santa Clara, CA, USA). The method used a binary gradient with mobile phases containing methanol and water acidified with 0.1% formic acid at a flow rate of 0.5 mL/min. The injection volume was 10 μL. Data acquisition was performed by Shimadzu LabSolutions software (version 1.20).

#### 2.5.4. Heart Rate Measurement

Heart rate (HR) measurements were recorded for 1 min (i.e., during the first and the last minute of relaxation session) by a high frequency infrared light earlobe sensor, worn by each participant on the left earlobe and further processed and presented for virtual reality (VR) application by a microcontroller. Each pulse duration was determined and recorded by identifying the intervals between the highest blood oxygen saturation points. Heart rate was calculated by the formula:

(1) $$\mathrm{HR}=\frac{60000}{{\mathrm{HR}}_{\mathrm{durr}}}$$

where HR_durr_ refers beat-to-beat intervals.

#### 2.5.5. Galvanic Skin Response

Galvanic skin response (GSR) was measured as a digitized resistance—the average of one-minute activity level. GSR was recorded for 1 min (i.e., during the first and the last minute of relaxation session) and calculated by the formula:

(2) $$\mathrm{GSR}=V_{\mathrm{in}}\;\cdot \;\left(\frac{V_{\mathrm{ref}}}{1024}\right)$$

where V_ref_ refers to the initial voltage and V_in_ refers to the output voltage.

Each subject’s cutaneous electrical resistance was measured via electrodes attached to the index and middle fingers of the person’s left hand. A 10-bit digital resolution and 5000 mV initial analog voltage (V_ref_) were chosen for digitization of measurements. The measured change in skin resistance changes the resistance ratio of the resistor network, which affects the voltage displayed by the sensor at the output (V_in_). This, in turn, is digitized, thus obtaining the mentioned 10-bit signal. Neutral resistor network matching (no human skin connected to the sensor system) was selected at the 3418 mV point (digital result—700). When the change in skin resistance causes a 4.9 mV change in the analog sensor output, the digital value changes to 1.

### 2.6. Statistical Analysis

Statistical analysis was performed with R version 3.6.0. Quantitative variables are presented as median (interquartile range) (IQR) or mean ± standard deviation (SD). The paired samples Wilcoxon test was used to analyze the differences in physiological and biochemical stress biomarkers before and after relaxation sessions. The paired samples *t*-test was used to evaluate differences in psychological stress measures (mood status, fatigue, and strain) before and after relaxation sessions. The impact of variation in pre-session values of physiological and biochemical stress measures was minimized by calculating the percentage change according to the following formula:

(3) $$\Delta \%=\frac{\left(\mathrm{post}-\mathrm{session}-\mathrm{pre}-\mathrm{session}\right)}{\mathrm{pre}-\mathrm{session}}\times 100.\;$$

One sample Wilcoxon test was employed to examine whether percentage changes in biochemical and physiological stress biomarkers in response to four distinct relaxation approaches are statistically significant. Moreover, the Friedman test for repeated measures was used to evaluate the differences in the influence of distinct relaxation techniques on percentage change in biochemical and physiological measures. The level of statistical significance was set at 0.05 for two-tailed testing.

## 3. Results

### 3.1. Perceived Stress and Anxiety Levels in the Study Sample

The analysis of PSS questionnaire showed that the majority of the study subjects considered their lives non-stressful with PSS values ≤ 13 (25.6%) or felt a moderate stress level (74.4%) with scores ranging from 14 to 26 during the previous month. The mean ± SD scores of T-Anxiety (39.98 ± 6.04) and S-Anxiety (36.12 ± 11.21) revealed that there were no clinically significant symptoms of anxiety in our study sample.

### 3.2. Influence of Distinct Relaxation Sessions on Subjectively Evaluated Mood Status, Fatigue, and Strain Level

The paired samples t-test showed significantly reduced strain level after all modalities of relaxation sessions. Similarly, electroencephalographic, respiratory, and galvanic skin response-based biofeedback relaxation sessions resulted in a decreased self-reported fatigue level. However, none of the tested relaxation strategies significantly improved subjects’ mood status (Table 2).

### 3.3. Influence of Distinct Relaxation Sessions on Salivary Glucocorticoid Levels, Heart Rate, and Galvanic Skin Response Values

All virtual-reality based relaxation techniques significantly decreased absolute concentration of salivary cortisol after electroencephalographic, galvanic skin response, and mindfulness-based biofeedback relaxation sessions. Moreover, administration of the biofeedback-supported respiratory relaxation session resulted in decline of absolute cortisol concentration, which was close to being statistically significant. Similarly, all relaxation techniques significantly (except for galvanic skin response biofeedback which approached a borderline level of statistical significance) reduced absolute cortisone and total salivary glucocorticoid (cortisol + cortisone) levels (Table 3).

Furthermore, galvanic skin response values were significantly increased by all biofeedback-assisted relaxation techniques. However, no significant differences in heart rate values were observed after four different relaxation approaches (Table 4).

### 3.4. Comparison of Modalities of Virtual Reality-Based Relaxation Techniques

**Figure 1:**
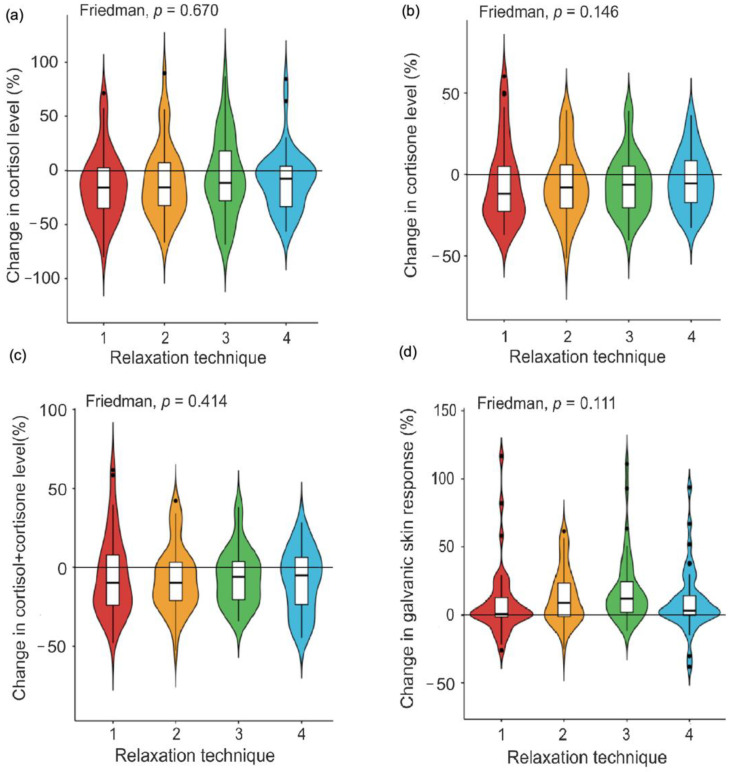
Percentage changes in biochemical (**a**–**c**) and physiological (**d**) stress biomarkers after distinct virtual reality-based relaxation sessions (1—EEG biofeedback, 2—Mindfulness-based biofeedback, 3—Respiratory biofeedback, 4—Galvanic skin response biofeedback).

Administration of all virtual reality-based relaxation techniques resulted in a decrease of salivary steroid hormone levels as indicated by negative percentage change values. Although differences in the percentage change of biochemical stress measures between distinct relaxation techniques were non-significant (cortisol: χ^2^ = 2.36, *p* = 0.670, cortisone: χ^2^ = 6.82, *p* = 0.146, cortisol + cortisone: χ^2^ = 3.94, *p* = 0.414) the highest statistically significant percentage change (median (IQR), one-sample Wilcoxon test) in salivary cortisol level was observed after mindfulness-based biofeedback (−15.59 (39.63), *p* = 0.032) while the largest percentage change in salivary cortisone and total glucocorticoid levels were seen after EEG the biofeedback-assisted relaxation session (−11.93 (27.49), *p* = 0.088, −9.76 (31.67), *p* = 0.081, respectively). Similarly, all relaxation techniques effectively increased galvanic skin response values (i.e., positive percentage change). The greatest statistically significant increase in galvanic skin response percentage change (median (IQR), one-sample Wilcoxon test) was found after the respiratory biofeedback relaxation session (11.99 (22.44), *p* = 1.374·10^−6^). However, the Friedman test for repeated measures revealed that differences in galvanic skin response percentage change among the four experimental relaxation sessions were non-significant (χ^2^ = 7.52, *p* = 0.111) (Figure 1).

## 4. Discussion

This study aimed to analyze the influence of brief virtual reality-based biofeedback-assisted relaxation techniques on psychological, physiological, and biochemical stress measures in healthy adults. Our results showed that all interventions led to significantly reduced subjectively perceived psychological strain level and diminished secretion of salivary glucocorticoids. Specifically, even a single application of relaxation techniques facilitated a decrease in biochemical stress measures including cortisol and cortisone levels, as well as total glucocorticoid output. The findings of other studies analyzing the efficacy of biofeedback-based training on salivary cortisol levels are inconsistent. For example, Kotozaki et al. [29] found that participants involved in four-week heart rate and cerebral blood flow control assisted biofeedback training demonstrated significantly reduced salivary cortisol concentration, compared with subjects engaged in regular life during that period. In contrast, no significant changes in salivary cortisol level were observed in the study conducted by Lemaire et al. [30] who investigated the effectiveness of 4-week stress-reduction intervention based on heart rate variability biofeedback in 40 physicians from various medical practices. Similarly, a study performed in 75 correctional officers showed no significant effect on total cortisol output evaluated over a 24-h period after a 3-month stress management program enhanced by heart rate variability coherence feedback [31]. These inconsistencies might be explained by methodological issues, since salivary cortisol level was used as the main outcome measure in the aforementioned studies where longer-term effect of biofeedback-aided relaxation training was investigated. It is suggested that salivary cortisol exhibits state-like properties with substantial day-to-day fluctuations caused by variations in mood, emotions, behavioral, and health indicators [32]. In the current study, we measured salivary glucocorticoid concentrations just before and immediately after the relaxation sessions. The short interval between sample collection with a relaxation session in between ensure that alterations in salivary cortisol and cortisone levels would be mainly influenced by the participation in virtual reality-based relaxation sessions rather than daily fluctuations or circadian rhythmicity. However, there is some evidence that biofeedback assisted stress management training is an effective tool for controlling stress. Bouchard et al. [33] showed that soldiers who were immersed in three-day heart rate and skin conductance biofeedback-assisted stress management practices demonstrated lower cortisol reactivity towards stressful live first aid simulation compared with participants who received the usual training offered to military personnel.

Other studies that investigated short-term effects of relaxation on salivary cortisol levels used non-biofeedback-assisted relaxation techniques. For example, a study conducted by Ventura et al. [34] analyzed the efficacy of 30-min relaxing interventions, including listening to relaxing music and reading decoration magazines in silence in a group of pregnant women awaiting amniocentesis. Results demonstrated a significant decrease in salivary cortisol concentration after relaxing interventions, especially listening to music, compared with the pregnant women allocated to the control group as they were simply sitting in the waiting-room with a relative or friend. In another study, distinct relaxation techniques such as progressive muscle relaxation, diaphragmatic breathing, and guided imagery, diaphragmatic breathing, and autogenic training, as well as a participant’s self-selected “favorite” relaxation strategy, contained within 120-min cognitive–behavioral stress management session were tested in the group of HIV-seropositive women. The results revealed a significant decrease in salivary cortisol level only after diaphragmatic breathing and guided imagery relaxation strategy, while other techniques had a very limited effect on HPA axis activity. Similarly, Pawlow et al. [35] aimed to analyze the effectiveness of 20-min abbreviated progressive muscle relaxation therapy in adults suffering from night eating syndrome. The results indicated significantly reduced cortisol secretion in subjects who attended muscle relaxation session compared with the control group participants who were quietly sitting alone in the room for 20 min. Taken together, these findings seem to suggest that even single-session brief relaxation interventions may effectively reduce HPA axis activity. Since previous studies examined these interventions in patients with specific disorders or subjects awaiting stressful events, our study confirms the effectiveness of brief virtual reality-based relaxation programs in healthy young and middle-aged adults experiencing daily hassles.

In addition to these benefits, all the virtual reality-based relaxation techniques that were tested led to reduced skin electrical conductance (i.e., increased galvanic skin response values), which indicates diminished sympathetic activity. Specifically, respiratory biofeedback-assisted relaxation session resulted in the greatest increase of galvanic skin response values. These findings support the results from previous research showing a significant decline in skin conductance level under resting conditions in subjects who underwent five weekly sessions of respiratory sinus arrhythmia biofeedback training [36]. Moreover, Strunk et al. [37] demonstrated that even a brief 5-min visual biofeedback-assisted relaxation session was effective in reducing electrodermal activity in a group of volunteers from psychology classes. The study also reported a potential placebo effect of biofeedback-aided relaxation since both accurate and false-feedback conditions generated physiological changes associated with relaxation response [37].

In the current study, none of the tested relaxation strategies were effective in reducing subjects’ heart rate values. Distinct findings were observed in previous research since some studies [19,38] showed a significant decrease in heart rate values while another [30] reported no significant alterations in heart rate measures from pre- to post-relaxation training. These inconsistencies might be due to differences in participants’ baseline heart rate values. Specifically, Dillon et al. [38] performed the Trier social stress test prior the administration of 30-min biofeedback aided relaxation training. In the study conducted by Lemaire et al. [30], as well as in the present study, subjects were not involved in the stress induction procedure and resting heart rate was used as a baseline measure. Thus, the effectiveness of a brief relaxation intervention in reducing heart rate values, an objective marker of arousal, is more evident among temporarily stressed individuals compared to subjects under resting conditions.

Although all relaxation techniques had a beneficial effect on individuals’ physiological and biochemical stress measures, no significant differences between the distinct approaches using different feedbacks were found. As relaxation sessions in the current study took only 12 min and all modalities of interventions had a positive effect, we might assume that VR as a realm for feedback has demonstrated high potential for stress reduction. This approach might be particularly suitable for individuals who are not capable of adhering to a strict and time-consuming stress management intervention schedule.

This study has several limitations that need to be addressed in future research. Firstly, participants with low and moderate perceived stress level were recruited in the current study. Thus, the effectiveness of virtual reality-based relaxation techniques should be confirmed in a population of highly stressed individuals. Secondly, the efficacy of distinct relaxation sessions was evaluated by measuring salivary glucocorticoid levels, heart rate, and galvanic skin response values. Since these measures represent the activity of the HPA axis and either both branches of the autonomic nervous system or only the sympathetic nervous system, there is a lack of a direct marker of parasympathetic nervous system activity such as frequency and time–domain heart rate variability measures. Thirdly, there was no control group which was not exposed to virtual reality-based relaxation methods. Thus, a study with no-intervention control group could be conducted to confirm the efficacy of virtual reality relaxation tools. Finally, we evaluated the short-term effect of brief relaxation sessions under carefully controlled laboratory conditions. Therefore, it would be worth testing longer-term impact of these relaxation strategies under real-life settings.

## 5. Conclusions

Single application of four tested virtual reality-based brief relaxation approaches induced a relaxation state manifested by diminished secretion of salivary glucocorticoids and decreased electrodermal activity, as well as reduced subjective evaluation of strain. Contrary to our primary hypothesis, no differences in the effectiveness of distinct virtual reality-based relaxation sessions were observed in the current study. Thus, relaxation techniques adapted to VR, namely based on EEG, GSR, breathing, and mindfulness biofeedback, all demonstrate a high potential for stress reduction and might be particularly suitable for individuals who are not capable of adhering to a strict and time-consuming stress management intervention schedule. Since the desired goal of biofeedback-based relaxation procedure is to achieve sustainable positive effect, our future research will be focused on the measurement and comparison of long-term changes in stress biomarkers in individuals who adhere to a particular VR-based relaxation technique.

## Figures and Tables

**Table 1:** Sociodemographic and lifestyle characteristics of the study sample.

Variable	Mean ± SD or *n* (%)
Gender	
Women	28 (65.0)
Men	15 (35.0)
Age (Years)	34.7 ± 7.2
Marital status	
Single	17 (39.5)
Married	21 (48.9)
Divorced	5 (11.6)
Education	
Secondary	1 (3.3)
Tertiary	42 (97.7)
Smoking status ^1^	
Non-smoker	31 (75.6)
Moderate smoker	4 (9.8)
Heavy smoker	6 (14.6)
Exposure to environmental tobacco smoke ^1^	
No	34 (82.9)
Yes	7 (17.1)
Physical activity at work ^1^	
Inactive	36 (87.8)
Active	5 (12.2)
Leisure time physical activity ^1^	
Inactive	14 (34.1)
Active	27 (65.9)

^1^*n* = 43 for all variables except for smoking status (*n* = 41), exposure to environmental tobacco smoke (*n* = 41), physical activity at work (*n* = 41), and leisure-time physical activity (*n* = 41).

**Table 2:** Effects of distinct virtual reality-based relaxation techniques on psychological stress measures.

Variable	Pre-Session (*n* = 43) Mean ± SD	Post-Session (*n* = 43) Mean ± SD	*p*-Value
Electroencephalographic biofeedback			
Mood	3.74 ± 0.79	3.79 ± 0.83	0.643
Fatigue	3.30 ± 1.12	3.67 ± 0.99	0.002
Strain	3.47 ± 1.08	3.84 ± 0.92	0.022
Mindfulness-based biofeedback			
Mood	3.93 ± 0.77	3.95 ± 0.72	0.743
Fatigue	3.56 ± 1.03	3.63 ± 1.09	0.596
Strain	3.88 ± 0.91	4.19 ± 0.79	0.005
Respiratory biofeedback			
Mood	3.67 ± 0.92	3.86 ± 0.77	0.088
Fatigue	2.91 ± 1.11	3.37 ± 1.05	4.229 × 10^−5^
Strain	3.42 ± 1.05	4.09 ± 0.87	1.941 × 10^−5^
Galvanic skin response biofeedback			
Mood	3.63 ± 0.90	3.70 ± 0.86	0.498
Fatigue	3.30 ± 1.06	3.63 ± 0.93	0.033
Strain	3.58 ± 1.07	3.95 ± 1.02	0.048

**Table 3:** Effects of distinct virtual reality-based relaxation techniques on the absolute concentrations of glucocorticoids in saliva.

Variable	Pre-Session (*n* = 43) Median (IQR)	Post-Session (*n* = 43) Median (IQR)	*p*-Value
Electroencephalographic biofeedback			
Cortisol (ng/mL)	0.98 (0.63)	0.96 (0.51)	0.010
Cortisone (ng/mL)	7.01 (4.44)	6.66 (2.54)	0.010
Cortisol + cortisone (ng/mL)	8.09 (5.28)	7.61 (2.89)	0.011
Mindfulness-based biofeedback			
Cortisol (ng/mL)	1.21 (0.81)	1.02 (0.77)	0.004
Cortisone (ng/mL)	7.36 (3.83)	7.18 (4.02)	0.020
Cortisol + cortisone (ng/mL)	8.67 (4.41)	8.36 (4.68)	0.007
Respiratory biofeedback			
Cortisol (ng/mL)	0.98 (0.67)	0.94 (0.61)	0.073
Cortisone (ng/mL)	6.97 (3.18)	6.55 (2.64)	0.009
Cortisol + cortisone (ng/mL)	7.95 (3.91)	7.57 (3.00)	0.010
Galvanic skin response biofeedback			
Cortisol (ng/mL)	1.26 (1.01)	0.95 (0.72)	0.010
Cortisone (ng/mL)	7.44 (4.39)	6.78 (3.91)	0.054
Cortisol + cortisone (ng/mL)	8.85 (5.24)	8.00 (4.58)	0.025

**Table 4:** Effects of distinct virtual reality-based relaxation techniques on physiological stress biomarkers.

Variable	Pre-Session (*n* = 43), Median (IQR)	Post-Session (*n* = 43), Median (IQR)	*p*-Value
Electroencephalographic biofeedback			
GSR	506 (202.5)	517 (177.5)	0.029
HR (bpm)	72 (12)	72 (16)	0.796
Mindfulness-based biofeedback			
GSR	472 (182.5)	521 (144.5)	4.631 × 10^−4^
HR (bpm)	72 (12)	73 (13)	0.1048
Respiratory biofeedback			
GSR	492 (162.5)	573 (84.5)	2.743 × 10^−6^
HR (bpm)	72 (12)	72 (15)	0.976
Galvanic skin response biofeedback			
GSR	504 (156)	544 (132)	0.003
HR (bpm)	74 (10)	73 (10)	0.272

## Data Availability

The data presented in this study are available on request from the corresponding author.
